# Integrative single-cell analysis of longitudinal t(8;21) AML reveals heterogeneous immune cell infiltration and prognostic signatures

**DOI:** 10.3389/fimmu.2024.1424933

**Published:** 2024-07-17

**Authors:** Xue-Ping Li, Jiang-Tao Song, Yu-Ting Dai, Wei-Na Zhang, Bai-Tian Zhao, Jia-Ying Mao, Yan Gao, Lu Jiang, Yang Liang

**Affiliations:** ^1^ Department of Hematologic Oncology, Sun Yat-sen University Cancer Center, Guangzhou, China; ^2^ State Key Laboratory of Oncology in South China, Guangdong Provincial Clinical Research Center for Cancer, Sun Yat-sen University Cancer Center, Guangzhou, China; ^3^ Shanghai Institute of Hematology, State Key Laboratory of Medical Genomics, National Research Center for Translational Medicine at Shanghai, Ruijin Hospital, Shanghai Jiao Tong University School of Medicine, Shanghai, China; ^4^ Department of Hematology, Guangzhou Women and Children’s Medical Center, Guangzhou, China; ^5^ Department of Medical Oncology, Sun Yat-sen University Cancer Center, Guangzhou, China

**Keywords:** single-cell technology, heterogeneity, T cells, AML, prognosis

## Abstract

**Introduction:**

Immunotherapies targeting T cells in solid cancers are revolutionizing clinical treatment. Novel immunotherapies have had extremely limited benefit for acute myeloid leukemia (AML). Here, we characterized the immune microenvironment of t(8;21) AML patients to determine how immune cell infiltration status influenced prognosis.

**Methods:**

Through multi-omics studies of primary and longitudinal t(8;21) AML samples, we characterized the heterogeneous immune cell infiltration in the tumor microenvironment and their immune checkpoint gene expression. Further external cohorts were also included in this research.

**Results:**

CD8+ T cells were enriched and *HAVCR2* and *TIGIT* were upregulated in the CD34^+^CD117^dim^%-High group; these features are known to be associated with immune exhaustion. Data integration analysis of single-cell dynamics revealed that a subset of T cells (cluster_2) (highly expressing *GZMB, NKG7, PRF1* and *GNLY*) evolved and expanded markedly in the drug-resistant stage after relapse. External cohort analysis confirmed that the cluster_2 T-cell signature could be utilized to stratify patients by overall survival outcome.

**Discussion:**

In conclusion, we discovered a distinct T-cell signature by scRNA-seq that was correlated with disease progression and drug resistance. Our research provides a novel system for classifying patients based on their immune microenvironment.

## 1 Introduction

Immunotherapies have had good effects against solid cancers by targeting immune evasion to restore T-cell immunity and are revolutionizing cancer treatment (1, 2). However, the development of immunotherapy for acute myeloid leukemia (AML), which is characterized by the blockade of myeloid differentiation in hematopoietic stem and progenitor cells, has been rather challenging (3).

AML cells can escape or inhibit immune system mechanisms by, for example, downregulating major histocompatibility complex (MHC) molecules or upregulating inhibitory ligands (4, 5). Treatment for AML depends largely on T-cell-mediated effects (4, 5). Exhausted T cells play a role in AML relapse, even for patients receiving allogeneic hematopoietic cell transplantation (6).

Deciphering the immunosuppressive microenvironment of AML has become a research hotspot (4) (7). Recently, the development and application of single-cell technology have allowed the characterization of tumor-infiltrating T cells across cancers (8–10). Notably, exhausted T cells within different cancer types are diverse and heterogeneous.

AML with t(8;21)(q22;q22.1), a special subtype of core binding factor carrying the *RUNX1*-*RUNX1T1* fusion genes, accouts for nearly 8% of all AMLs (11). Though normally considered as a favorable subtype, nearly 30% of patients with t(8;21)AML relapse clinically (12, 13).

Our group has been dedicated to evaluating the heterogeneity of t(8;21) AML. We have constructed a multi-omics dataset for studying t(8;21) AML; this dataset includes single-cell RNA-seq as well as whole-exome sequencing data (14–16). In our previous multi-omics studies (14, 15), patients with relapsed or refractory disease were shown to have a greater proportion of CD34^+^CD117^dim^ cells (Blast population 1, BP1) than other patients.

Here, we aimed to apply a multi-omics approach to characterize the heterogeneity of immune microenvironment of t(8;21) AML patients and determine how immune cell infiltration status influences prognosis.

## 2 Methods

### 2.1 Study design

The study design is illustrated in **Supplementary Figure S1**. Briefly, we obtained data from newly diagnosed t(8;21) AML patients, including single-cell RNA-seq and bulk RNA-seq data (National Omics Data encyclopedia (NODE), OEP000629), from our previous reports (14). Patients received standard “3 + 7” induction regimens, including idarubicin and cytarabine, followed by high-dose cytarabine-based consolidation or hematopoietic stem cell transplantation (HSCT). In total, 28 of 62 t(8;21) AML patients relapsed. Of these 28 relapse patients, we observed 20 patients with drug-resistant disease stages (DRD).

We also used gene expression data of AML patients generated from Affymetrix arrays and RNA-seq. Data from the GSE37642 dataset, which included data obtained via the GPL96 (n=417) and GPL570 (n=136) platforms, and the GSE106291 (GPL18460) (n=250) dataset were downloaded online (17–20). We also downloaded the Beat AML (n = 200) (21) and TCGA LAML (n=140) (22) datasets. Patients were treated according to protocols described in the corresponding literature. In summary, data from 1205 patients were analyzed in this study (**Table 1**).

**Table 1:** The gene sets analyzed in this study.

Patients	Samples	Platform	Accession number
t(8;21) AML	BMMC	10xGenomics single-cell	NODE_OEP000629 (14)
t(8;21) AML	BMMC	bulk RNA-seq	NODE_OEP000629 (14)
AML	BMMC	GPL96, GPL570	GSE37642 (17–20)
AML	BMMC/PBMC	GPL18460	GSE106291 (17)
AML	BMMC	bulk RNA-seq	TCGA_LAML (22)
AML	BMMC	bulk RNA-seq	Beat AML (21)

### 2.2 Single-cell RNA-seq and bulk RNA-seq analysis

Bone marrow mononuclear cells (BMMCs) from t(8;21) AML patients were collected and subjected to the 10X Chromium platform for indexed sequencing of libraries following paired-end (2 × 150 bp) sequencing on a NovaSeq platform (Illumina) to conduct the single-cell RNA-seq analysis. The detailed process was described in our previous research (14, 15). To generate gene expression matrices, we utilized the cellranger tool (version 3.1.2, default settings) from 10X Genomics to align single-cell RNA sequencing (scRNA-seq) data with the human GRCh38 reference genome (2020-A version). Both the cellranger software and the GRCh38 reference were downloaded from the 10X Genomics website (https://www.10xgenomics.com). Subsequently, the gene expression matrices were imported into the Seurat package in R for further analysis (23). For quality control, cells expressing fewer than 800 genes or containing more than 10% mitochondrial RNA were excluded. The resulting filtered count matrices from different time points were then merged using the Seurat package. The expression data underwent normalization through a global-scaling normalization method, as implemented by default in the Seurat package. Following normalization, 2000 variable genes were identified to analyze expression variability across samples. Batch effects were removed using ComBat (24, 25). The uniform manifold approximation and projection (UMAP) technique was used for visualization (26). Cell cycle phase scores were calculated using the built-in function CellCycleScoring in Seurat with default parameters (27). Cells were annotated utilizing the machine-learning-based software SingleR (28), alongside the identification of high expression levels of canonical markers, such as CD34 for progenitor cells, CD3 for T-cells, CD79A for B cells, and CA1 for erythroid cells. For T-cell cluster analysis, unsupervised clustering was conducted on the T-cells derived from t(8;21) AML samples. This was performed using the “FindClusters” function in Seurat with the resolution parameter set to 0.8. Following this, five distinct T-cell clusters were identified. Marker genes for each cluster were determined through the “FindAllMarkers” function, adhering to criteria that included a log2(fold change) greater than 0.58 (fold change > 1.5), min.pct > 0.1, and adjusted P < 0.05.

The raw bulk RNA-seq data from t(8;21) AML patients at diagnosis were obtained previously. Briefly, after aligning raw reads to human reference genome hg19, we generated indexed BAM files with SAMtools and acquired count matrix with DESeq2 (29, 30). To calculate the gene expression, we used the fragments per kilobase million after nomalization. In addtion, limma package (31) was applied to identify differentially expressed genes. A heatmap of the 79 immune checkpoint genes was generated via the Hiplot platform (https://hiplot.com.cn/).

### 2.3 Cell-cell communication analysis

CellPhoneDB (http://www.cellphonedb.org/) (32) was applied to analyze cell-cell communication between different cell types with default parameters. Mean expression of each receptor-ligand pair was calculated and enrolled for further analysis.

### 2.4 Construction of the T-cell signature from cluster_2 T cells via scRNA-seq

Overexpressed genes in cluster_2 T cells were defined as those with an average log2(fold change) greater than 1.0 and a *p* value less than 0.05 in the scRNA-seq data. A total of 178 genes were ultimately screened out from among the highly expressed genes in the scRNA-seq data of cluster_2 T cells (**Supplementary Table S1**). GSE37642 (German AMLCG 1999) dataset was applied as the training dataset. We first conducted univariate cox regression analyis to determine the significantly prognostic genes in cluster_2 T cells. Then, least absolute shrinkage and selection operator (LASSO) regression analysis was utilized to calculate the parameters of lambda.min and lambda.1se, which providing the optimal gene number interval to construct the model. Afterwards, we performed the multi-variate Cox regression to determine the final risk model, of which 14 genes with high coefficients were selected, 14 T-cell-related genes (named the 14TGS).

### 2.5 Immune cell infiltration

QuanTIseq (33), CIBERSORT (34) and EPIC (35) were used to estimate the proportions of infiltrated immune cell types in the bone marrow microenvironment of t(8;21) AML patients. Groups were compared with the two-sided Wilcoxon rank-sum test.

### 2.6 Functional enrichment analysis

Kyoto Encyclopedia of Genes and Genomes (KEGG) (36) and Gene Ontology (GO) (37) enrichment analyses were applied to the 178 genes that characterized the cluster_2 T cells.The adjusted Benjamini-Hochberg *P* value was calculaeted to compare groups.

### 2.7 Statistical analysis

Kaplan–Meier plots were generated, and *P* values were determined via the log-rank test. Receiver operating characteristic (ROC) curves were analyzed with the timeROC package to calculate the areas under the ROC curves (AUCs) (38). R software (version 4.0.2) was used to perform the analysis.

## 3 Results

### 3.1 Heterogeneous immune infiltration profiles of the CD34^+^CD117^dim^%-high subgroup

To determine the landscape of infiltrating immune cells, we first conducted immune infiltration analysis with bulk RNA-seq data from patients newly diagnosed with t(8;21) AML (*n* = 62) in our cohort. The proportion of CD34^+^CD117^dim^ cells in the total CD34^+^ population of the BM was determined previously by multiparameter flow cytometry at diagnosis (14). Patients then received standard induction chemotherapy as described (14). Here, we dichotomized the whole cohort into two subgroups around the median proportion of CD34^+^CD117^dim^ cells (45.5%).

To estimate the proportions of various immune cell subsets, we used multiple methods, including quanTIseq (33), CIBERSORT (34) and EPIC (35). The immune infiltration results from quanTIseq showed that the CD34^+^CD117^dim^%-High subgroup had more CD8+ T cells and monocytes than the CD34^+^CD117^dim^%-Low subgroup (**Supplementary Figure S2A**). The EPIC and CIBERSORT algorithms also indicated a higher infiltration of CD8+T cells in the CD34^+^CD117^dim^%-High subgroup, though the P values were only 0.055 and 0.054, respectively (**Supplementary Figures S2B, C**). For the other infiltrating immune cells, we did not observe intergroup differences by these three methods. Thus, we further focused on the role of infiltrating CD8+ T cells, which might indicate a dysfunctional status of the T cells in the CD34^+^CD117^dim^%-High subgroup.

### 3.2 Overexpression of immune exhaustion-related genes in the CD34^+^CD117^dim^%-high subgroup

Immune checkpoint genes regulate the immune response by stimulating or inhibiting pathways (39) [for a summary of immune checkpoint genes, see (40)]. We hypothesized that CD8+ T cells in the CD34^+^CD117^dim^%-High subgroup were dysfunctional. We then analyzed the 79 immune checkpoint genes that function as active, inhibitory or two-sided immune chekcpoint genes (40), most of which were receptors or ligands in immune-related pathways.

**Figure 1:**
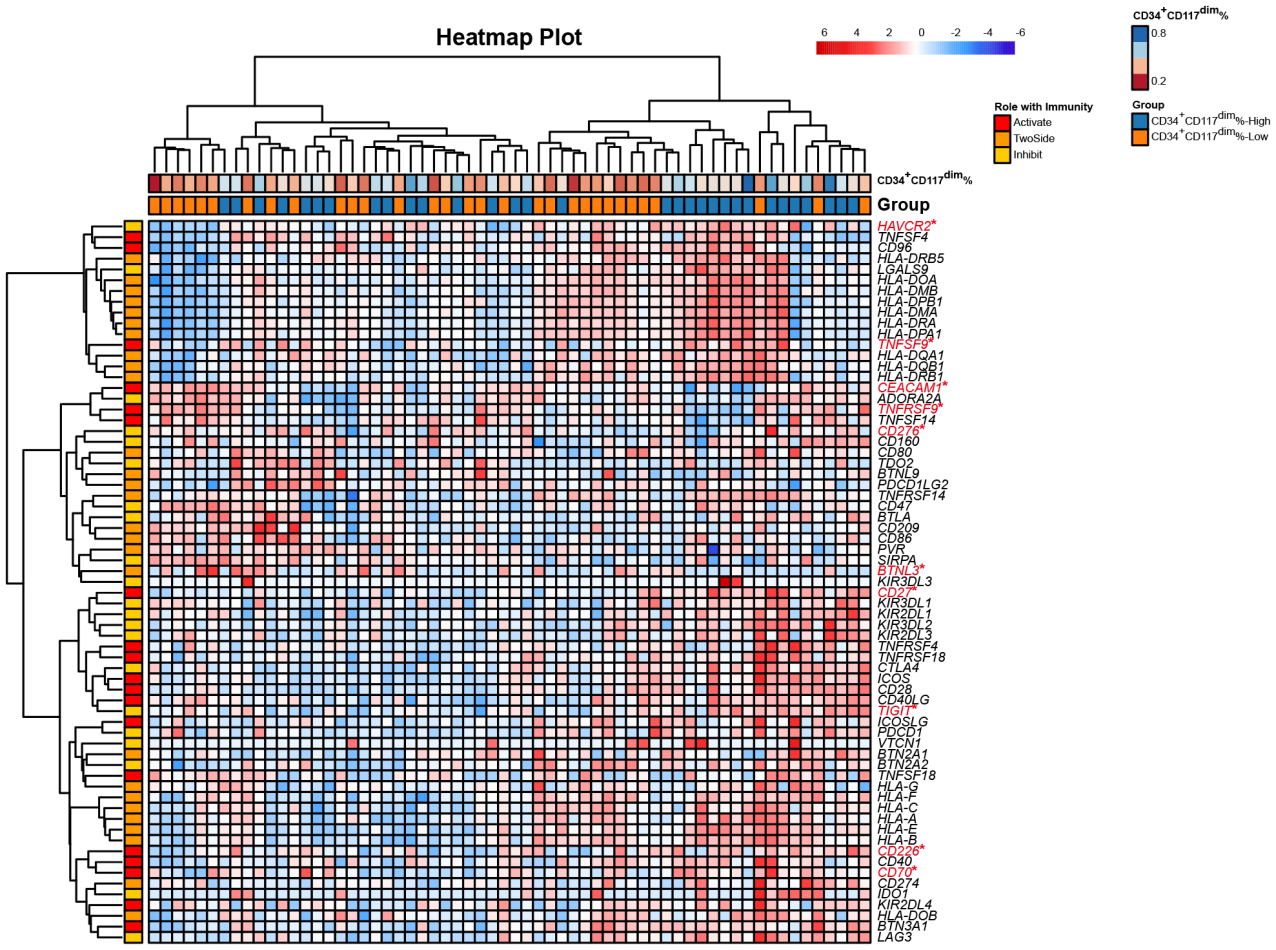
Heatmap of the expression of 79 immune checkpoint genes from bulk RNA-seq of t(8;21) AML patients at diagnosis. Each column represents a sample, and each row represents an immune checkpoint gene. The 79 genes were characterized as having active, inhibitory and two-sided effects on the immune responses. The upper row shows the corresponding percentage of CD34^+^CD117^dim^ cells in each sample detected via flow cytometry. The lower row shows the corresponding subgroup separated by the median CD34^+^CD117^dim^ population, namely CD34^+^CD117^dim^%-High subgroup and CD34^+^CD117^dim^%-Low subgroup. *Denotes the differentially expressed genes between the two subgroups, namely CD34^+^CD117^dim^%-High subgroup and CD34^+^CD117^dim^%-Low subgroup. Statistical analyses were conducted with a two-sided Wilcoxon rank-sum test. *p < 0.05.

The CD34^+^CD117^dim^%-High risk subgroup had a differential expression profile than the CD34^+^CD117^dim^%-Low risk subgroup had (**Figure 1**). Notably, the CD34^+^CD117^dim^%-High subgroup expressed high levels of immune checkpoint inhibitors, including *HAVCR2* (encoding the TIM-3 protein) and *TIGIT* (encoding the immunoglobin protein), which were known to be associated with immune exhaustion (**Figure 1**; **Supplementary Figure S3**). In addition, *CD27*, *CD70*, *CD226*, *CD276* and *TNFSF9* were also more highly expressed in the CD34^+^CD117^dim^%-High subgroup. On the other hand, *CEACM1* and *TNFRSF9* were more highly expressed in the CD34^+^CD117^dim^%-Low subgroup (**Supplementary Figure S3**). The highly immunosuppressive phenotype of the microenvironment might play an important role in promoting inferior outcomes in the CD34^+^CD117^dim^%-High subgroup.

### 3.3 Single-cell RNA-seq atlas of dynamic T-cell subsets in t(8;21) AML

To elucidate the complexity of the classifications and functions of T cells, we reanalyzed our previously generated scRNA-seq datasets of t(8;21) AML patients at different stages of disease progression, including the diagnosis, relapse and post-relapse drug-resistant stages (14). The t(8;21) AML patient received standard “3 + 7” induction chemotherapy to achieve complete remission (CR) followed by 3 courses of cytarabine consolidation. Relapse was observed, and then the patient was treated with IA combined with dasatinib which failed to achieve a CR (drug-resistant disease stage, DRD).

Integrated analysis of T cells at 3 time points revealed 5 subtypes of T cells (**Supplementary Figures S4A, B**). Cells were annotated utilizing the machine-learning-based software SingleR (28), verifying the identity of T cells (**Supplementary Figure S4C**). Compared to the rest BMMCs, T cells had a higher expression of T-cell markers, including *CD3D*, *CD3E* and *CD7*, each subtype had a unique gene signature (**Supplementary Figures S4D, E**).

**Figure 2:**
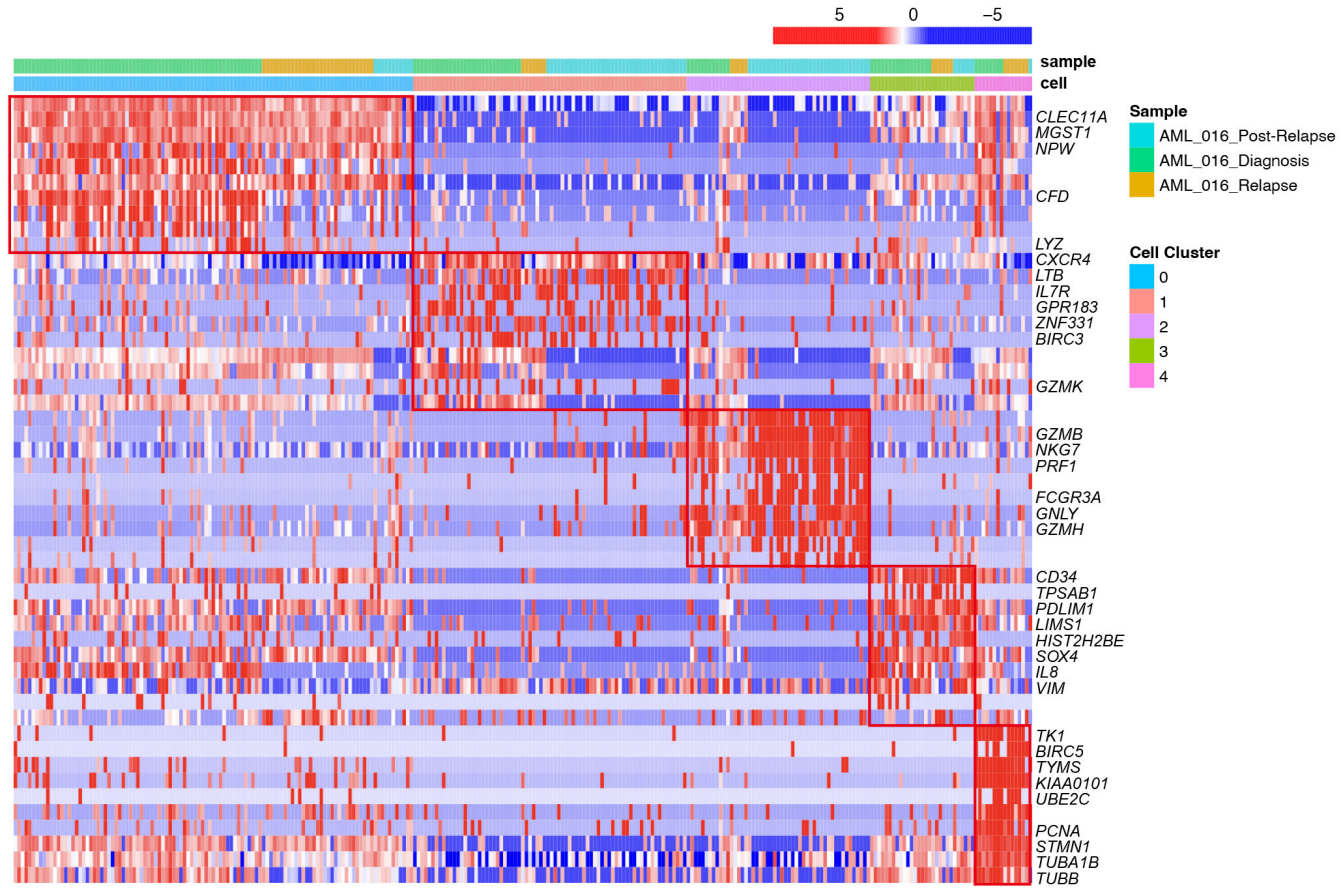
Heatmap of selected top differentially expressed marker genes for each of the 5 T-cell clusters across different stages, including diagnosis, relapse and post-relapse, according to the single-cell RNA-seq data. The upper row shows the corresponding sample from different stages. The lower row shows the cell clusters. Each row represents the relative expression level of genes, and each column represents a cell from the scRNA-seq data.

**Figure 3:**
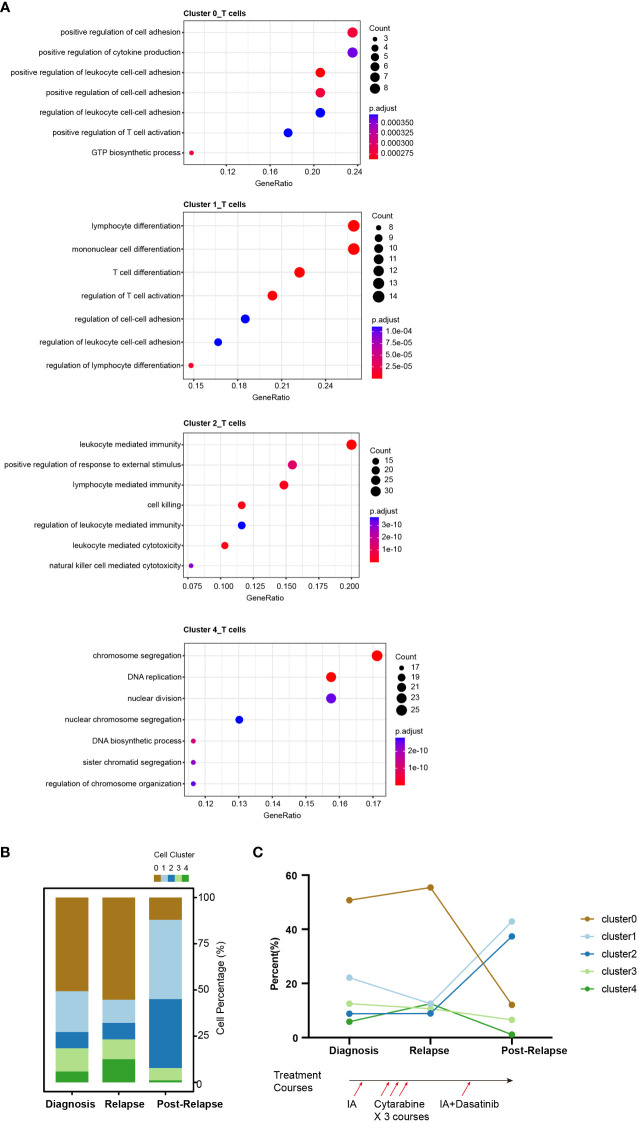
Single-cell RNA-seq atlas of T cells across disease states in the AML-016. **(A)** Gene Ontology enrichment analysis of the highly expressed genes in T cell clusters across disease stages. Biological process (BP) terms are shown, and the *P* value was adjusted by Benjamini-Hochberg correction. **(B)** Bar plot showing the relative proportions of the T-cell clusters across the disease samples. Each color represents a different T cell cluster. **(C)** Line chart showing the dynamic changes in the proportions of T-cell clusters across the disease samples. The lower panel shows the treatment course of AML-016. Briefly, patient received standard “3 + 7” induction chemotherapy to achieve complete remission (CR) followed by 3 courses of cytarabine consolidation. Relapse was observed, and then the patient was treated with IA combined with dasatinib which failed to achieve a CR.

To identify the subsets of T cells, we compared the expression of each gene between the clusters and the average of the other cells. We also conducted a functional enrichment analysis of the highly expressed genes in each cluster with Gene Ontology. Specifically, *LYZ*, *CLEC11A*, *MGST1* and *IFITM3* were highly expressed in cluster_0 (**Figure 2**). GO enrichment analysis showed that cluster 0 was enriched in the regulation of cell adhesion and T cell activation (**Figure 3A**). The genes overexpressed in cluster_1 were *LTB*, *IL7R* and *CXCR4*, which are markers of naïve T cells. Cluster_1 T cells were enriched in the T cell differentiation and lymphocyte differentiation (**Figure 3A**).

Cluster_2 highly expressed *GZMB*, *NKG7*, *PRF1* and *GNLY*, which are cytokines and effector molecules (**Figure 2**). Leukocyte-mediated immunity and cell killing were enriched in cluster_2 T cells. Cluster_3 highly expressed *CD34*, *IL8* and *SOX4*, which are required for T-cell development (**Figure 2**). Cluster_4 highly expressed *KIAA0101*, *TYMS*, *TK1* and *PCNA*, which are involved in the cell cycle, as further demonstrated by its higher G2M and S scores (**Supplementary Figure S4F**). DNA replication was enriched in cluster_4 T cells (**Figure 3A**).

We observed dynamic changes in the proportions of T-cell subtypes as the disease progressed and after treatment with multiple chemotherapies (**Figures 3B, C**). Specifically, cluster_2 T cells made up a low percentage of cells at disease onset and relapse, while in samples from patients who presented resistance to cytarabine treatment, cluster_2 T cells exhibited marked expansion, which may suggest that this T subtype reflected treatment failure and that the number of T cells contributed to drug resistance (**Figure 3C**).

### 3.4 Construction of the T-cell signature from cluster_2 T cells via scRNA-seq

AML cells interact with the immune microenvironment, including dysfunctional T cells and the accumulation of macrophages, further influencing patient prognosis and clinical outcome (4). We thus hypothesized that in AML patients, dysfunctional T cells existed and impaired survival. To test the potential prognostic value of the cluster_2 T-cells signature in the AML cohort, we constructed a T-cell signature. According to the criteria described in the Methods section, a total of 178 genes were included (**Supplementary Table S1**).

**Figure 4:**
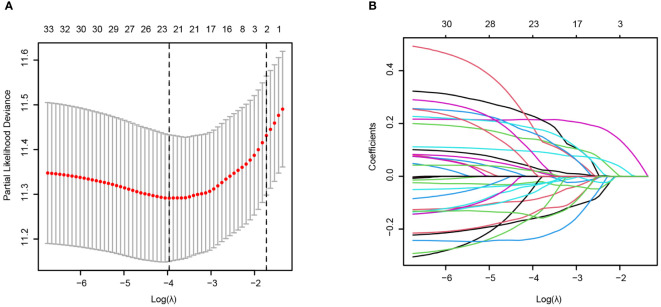
Construction of the T-cell signature from cluster_2 T cells via scRNA-seq based on LASSO regression in the training cohort (AMLCG 1999, GSE37642_GPL96). **(A)** The cross-validation results for determining the optimum value of log(λ). The dased line showed the value of lamda.min (Left) and lambda.1se (Right). **(B)** LASSO coefficients of the T-cell cluster model in the training cohort. Different curve denoted different genes.

To identify T-cell-related genes related to the prognosis of AML patients, we used the GSE37642_GPL96 (German AMLCG 1999) dataset as the training dataset. By means of univariate Cox regression, 34 of 178 genes were prognostic factors associated with overall survival (**Supplementary Table S2**). We further utilized LASSO regression to calculate the weighting coefficient at the optimal parameter λ (**Figures 4A, B**).

Using multivariate Cox regression analysis, a final risk model based on these 14 T-cell-related genes [*AES*, *DDIT4*, *GPR56* (*ADGRG1*), *HOPX*, *IFITM1*, *IFITM2*, *LAIR2*, *LSP1*, *MGEA5* (*OGA*), *OPTN*, *PRKCH*, *SH3BGRL3*, *SUN2* and *YWHAQ*] was constructed, which we named 14TGS (**Supplementary Figures S5**, **S6**).

**Figure 5:**
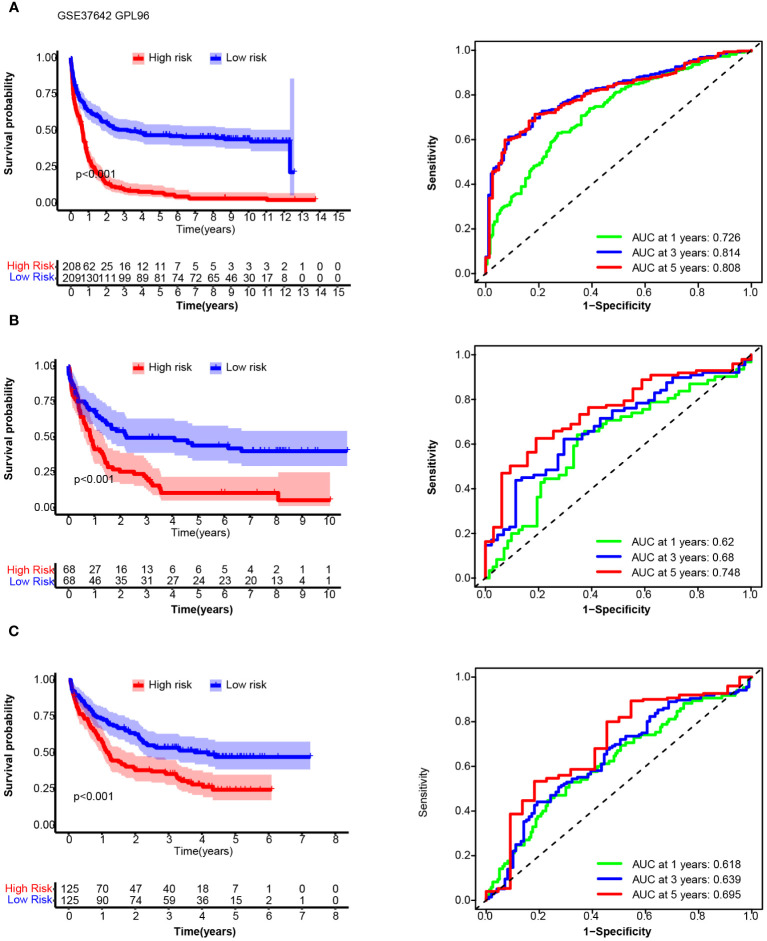
Overall survival (OS) and receiver operating characteristic (ROC) curve analysis of the 14TGS model in the training cohort (AMLCG 1999, GSE37642_GPL96, **(A)**, validation cohort-1 (GSE37642-GPL570, **(B)** and validation cohort-2 (GSE106291, **(C)**. (Left panel) Kaplan-Meier curves and log-rank tests were used to estimate and compare the survival differences between the high- and low- risk subgroup. (Right panel) Specificity and sensitivity of the 14TGS model was calculated using the timeROC package and aera under (AUC) at 1-year, 3-year, 5-year were shown.

We chose the median 14TGS score as the cutoff value to divide the whole group into a high-14TGS subgroup and a low-14TGS subgroup. In the training cohort, the high-14TGS subgroup had inferior outcomes, with a median OS time of 0.658 years vs. 3.181 years in the low-14TGS subgroup (**Figure 5A**). The area under the curve (AUC) values of the ROC curves for the prediction of 1-year, 3-year and 5-year OS were 0.726, 0.814 and 0.808, respectively (**Figure 5A**).

### 3.5 Expanded external cohorts to validate the 14TGS signature

Next, we tested the prognostic efficiency of 14TGS in external cohorts, including expression profiling by array (17–20), data generated with bulk RNA-seq, the TCGA dataset and the Beat AML dataset (21, 22).

In validation cohort_1 (GSE37642_GPL570), the median OS times in the high and low subgroups were 0.866 years and 2.236 years, respectively (log rank test, *p* < 0.001) (**Figure 5B**). The AUCs for predicting 1-year, 3-year and 5-year OS were 0.62, 0.68 and 0.748, respectively (**Figure 5B**). In validation cohort_2 (GSE106291), the median OS times in the high and low subgroups were 1.20 years and 4.19 years (log rank test, *p* < 0.001), respectively. The AUCs for the prediction of 1-year, 3-year and 5-year OS were 0.618, 0.639 and 0.695, respectively (**Figure 5C**).

**Figure 6:**
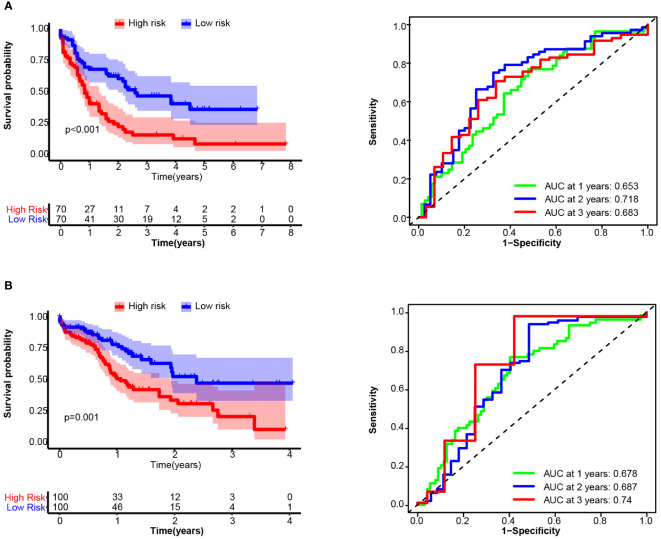
Overall survival (OS) and receiver operating characteristic (ROC) curve analysis of the 14TGS model in TCGA_LAML **(A)** and validation cohort-4 (Beat AML, **B**) (Left panel) Kaplan-Meier curves and log-rank tests were used to estimate and compare the survival differences between the high- and low- risk subgroup. (Right panel) Specificity and sensitivity of the 14TGS model was calculated using the timeROC package and aera under (AUC) at 1-year, 2-year, 3-year were shown.

14TGS had good performance in terms of gene expression according to the array platform, and we further tested the prognostic value of the data generated via the bulk RNA-seq platform. In validation cohort_3 (TCGA), the median OS times in the high and low subgroups were 0.838 years and 2.589 years (log rank test, *p* < 0.001), respectively. The AUCs for the prediction of 1-year, 2-year and 3-year OS were 0.653, 0.718 and 0.683, respectively (**Figure 6A**). In validation cohort-4 (Beat AML), the median OS times in the high and low subgroups were 1.06 years and 2.38 years (log rank test, *p* = 0.001), respectively. The AUCs for the prediction of 1-year, 2-year and 3-year OS were 0.678, 0.687 and 0.74, respectively (**Figure 6B**). In addition, we compared the 14TGS score among the different risk subgroup stratified by European LeukemiaNet (ELN) recommendations (41). As shown in **Supplementary Figure S7**, the adverse subgroup had the highest 14TGS score than favorable and intermediate subgroup, both for the TCGA AML and Beat AML datasets.

Consistent with the results in the training cohort, patients with high 14TGS scores presented poorer clinical outcomes than those with low 14TGS scores. All the AUC values supported the prognostic value of the 14TGS model.

## 4 Discussion

Our previous multiomics studies identified a unique cell subset, namely CD34^+^CD117^dim^ cells (Blast population 1, BP1) that promoted disease progression in t(8;21) AML. In this work, we characterized the special immune microenvironment of high risk t(8;21) AML patients (CD34^+^CD117^dim^%-High subgroup) and evaluated longitudinal dynamic changes in the expression profiles of immune cells. Our research further characterized the complex microenvironment of AML, including malignant blast populations and complex T-cell subsets leading to clinical relapse and an unresponsive state to chemotherapy. In addition, we constructed the 14TGS model to stratify AML patients by their predicted survival outcomes.

The mechanism by which dysfunctional T cells impact the chemotherapy response is rather complex (42). First, AML induces a largely immunosuppressive bone marrow microenvironment, as indicated by reduced levels of cytotoxic T cells and NK cells, and this immunosuppressive state facilitates the immune escape of leukemic stem cells (LSCs), leading to chemotherapy resistance (4). Other reports have shown that the increase in senescent-like T cells in AML is associated with a weaker reponse to induction chemotherapy (43).

T-cell exhaustion, characterized by the expression of inhibitor proteins, including PD-1 and TIM3, is a phenotypically and mechanistically distinct state of T cells (44). The presence of exhausted T cells is usually related to poor outcomes in patients with a variety of cancers (45). Several studies have described the heterogeneity and functions of T cells in AML. *Van Galen* et al. reported a reduction in the proportion of CTLs in AML patients compared to that in healthy controls (46), and *Schnorfeil* et al. reported an increase in PD-1 expression on T cells in the bone marrow at relapse posttransplantation (47). Additionally, the efficacy of immunotherapies, such as immune checkpoint inhibitors, is largely dependent on the levels of “precursor exhausted” CD8+ T cells, which have been suggested to exist according to studies of multiple tumor models (48–50).

In this study, we identifed 5 clusters of T cells. There was a high proportion of cluster_0 T cells (which highly expressed *LYZ*, *CLEC11A*, *MGST1* and *IFITM3*) at diagnosis and relapse, but this proportion declined sharply in the drug-resistant disease stages. Research has shown that *LYZ* (lysozyme) and *IFITM3* are important for antioxidant and anti-inflammatory effects (51, 52). This was in accordance with the phenomenon that the reduction of this cluster was drug resistant. Cluster_4 T cells (highly expressing *KIAA0101*, *TYMS*, *TK1* and *PCNA*), reflecting the state of cell proliferation rate, were reduced to an extremely low level. This finding was also consistent with the findings in another research analyzing the immune environment of melanoma (53), showing that the number of cycle-stage T cells decreased compared to that in the pre-treatment stage.

Cluster_1 T cells (which highly express *LTB*, *IL7R* and *CXCR4*) showed a unique dynamic pattern; compared with those at diagnosis, these cells decreased in number during relapse but increased in number during drug-resistant disease stages. Considering that these genes are involved in chemokine responses (54), this indicated a higly unstable inflammatory state.

Cluster_2 T cells, which highly expressed *GZMB*, *NKG7*, *PRF1* and *GNLY*, expanded rapidly and greatly following disease progression. Given that the expansion of cluster_2 T cells had no clinical response to reinduction therapy at the post-relapse stage. Cluster_2 T cells should not be characterized as cytotoxic T cells. Further *in vitro* experiments, which directly analyze the interaction between cluster_2 T cells and blasts, are still needed to clarify their role in disease progression.

The gene signature we constructed included 14 genes, namely, *AES* (*TLE5*), *DDIT4*, *GPR56* (*ADGRG1*), *HOPX*, *IFITM1*, *IFITM2*, *LAIR2*, *LSP1*, *MGEA5* (*OGA*), *OPTN*, *PRKCH*, *SH3BGRL3*, *SUN2* and *YWHAQ*. Most of these genes, including those encode *IFITM* proteins and leukocyte-associated immunoglobulin-like receptors, function in immune-related processes. The prognostic efficiency of 14TGS was further validated in other AML cohorts, and 14TGS demonstrated robust predictive value. Interestingly, one recent study published in *Blood* reported a distinct single-cell T-cell signature associated with stem cell transplantation outcome in AML patients; one of the included genes was *GPR56*, a biomarker of alloreactive CD8+ T cells (55). This further suggested that cluster_2 T cells are a novel subset that has different phenotypes and mechanisms of action.

In conclusion, through a multi-omics study of longitudinal t(8;21) AML, we revealed the heterogeneity of immune cells in the microenvironment and described the gene signatures and dynamic evolution of T-cell subsets. Further exploration of the gene signature of cluster_2 T cells revealed that this signature was a valuable prognostic indicator of survival in AML cohorts; the genes in this signature may represent clinical targets that could be investigated in more depth. Our research provides a novel system for classifying patients based on their immune microenvironment.

## Data availability statement

The original contributions presented in the study are included in the article/**Supplementary Material**. Further inquiries can be directed to the corresponding authors.

## Ethics statement

Ethical approval was not required for the study involving humans in accordance with the local legislation and institutional requirements. Written informed consent to participate in this study was not required from the participants or the participants’ legal guardians/next of kin in accordance with the national legislation and the institutional requirements.

## Author contributions

X-PL: Conceptualization, Data curation, Funding acquisition, Investigation, Writing – original draft, Writing – review & editing. J-TS: Data curation, Methodology, Writing – original draft. Y-TD: Data curation, Methodology, Resources, Software, Writing – original draft. W-NZ: Methodology, Resources, Writing – review & editing. B-TZ: Methodology, Resources, Writing – review & editing. J-YM: Methodology, Writing – review & editing. YG: Investigation, Writing – original draft, Writing – review & editing. LJ: Writing – original draft, Writing – review & editing. YL: Writing – original draft, Writing – review & editing.

## Supplementary material

Supplementary Table 1 Overexpressed genes in the cluster_2 T cells based on the single-cell RNA-seq data of AML-016. Genes were defined as those with an average log2(fold change) greater than 1.0 and a *p* value less than 0.05 in the scRNA-seq data. A total of 178 genes were ultimately screened out.

Supplementary Table 2 Univariate Cox analysis of the cluster_2 T genes to assess genes significantly correlated with overall survival in the training cohort (AMLCG 1999, GSE37642_GPL96). HR, hazard ratio.

Supplementary Table 3 LASSO Cox multivariate regression analysis to construct the risk model in the training cohort (AMLCG 1999, GSE37642_GPL96). 14 genes with high coefficients were selected and constitude the 14 T-cell-related genes (named the 14TGS). HR, hazard ratio. Coef, Coefficient.

Supplementary Figure 1 Overview of the study design.

Supplementary Figure 2 Infiltrating immune cells in the BM microenvironment of t(8;21) AML patients. Bar plot showing the comparison of inferred infiltrated immune cells between the two subgroups, namely CD34^+^CD117^dim^%-High subgroup and CD34^+^CD117^dim^%-Low subgroup, through quanTIseq **(A)**, EPIC **(B)** and CIBERSORT **(C)**. Statistical analysis was compared with a two-sided Wilcoxon rank-sum test.

Supplementary Figure 3 Differences in the expression of immune checkpoint genes between the two subgroups, namely CD34^+^CD117^dim^%-High subgroup and CD34^+^CD117^dim^%-Low subgroup. Statistical analysis was compared with a two-sided Wilcoxon rank-sum test.

Supplementary Figure 4 Single-cell RNA-seq atlas of T cells across disease states in the AML-016 cohort. **(A, B)**. Uniform manifold approximation and projection (UMAP) plot of T cells analyzed via scRNA-seq and integrated across all samples after removal batch effects. **(A)** T-cell clusters annotated with different colors. **(B)** Sample information annotated with different colors. **(C)** UMAP plot showing annotation results according to the the machine-learning-based software SingleR. **(D)** Violin plot showing the expression of T-cell markers, including *CD3D*, *CD3E*, *CD7*, between T cells and the rest of BMMCs in AML-016. **(E)** UMAP plot showing the expression of T-cell markers, including *CD3D*, *CD3E*, *CD7* and *RORA.* **(F)** Violin plot showing the scores of cell cycle-related genes, namely G2M and S scores. **(G)** CellPhoneDB analysis showed the potential communication of cluster_2 T cells with AML blasts.

Supplementary Figure 5 Forest plot showing the multivariate analyses in the training cohort (AMLCG 1999, GSE37642_GPL96). HR, hazard ratio. CI, confidence interval.

Supplementary Figure 6 Kaplan-Meier curves showed the survival difference stratified by T-cell-related genes in the training cohort (AMLCG 1999, GSE37642_GPL96). Log-rank tests were used to compare the survival differences.

Supplementary Figure 7 Comparsion of 14TGS score among different risk subgroup according to the ELN risk stratification. Wilcoxon test was performed to calculate the statistical differences. **p* < 0.05; ***p* < 0.01; ****p* < 0.001.
