# Supplementary figures and images for: Integrative single-cell analysis of longitudinal t(8;21) AML reveals heterogeneous immune cell infiltration and prognostic signatures
Source: Front Immunol. 2024 Jul 17;15:1424933. doi: 10.3389/fimmu.2024.1424933 (PMC11288856; doi:10.3389/fimmu.2024.1424933)

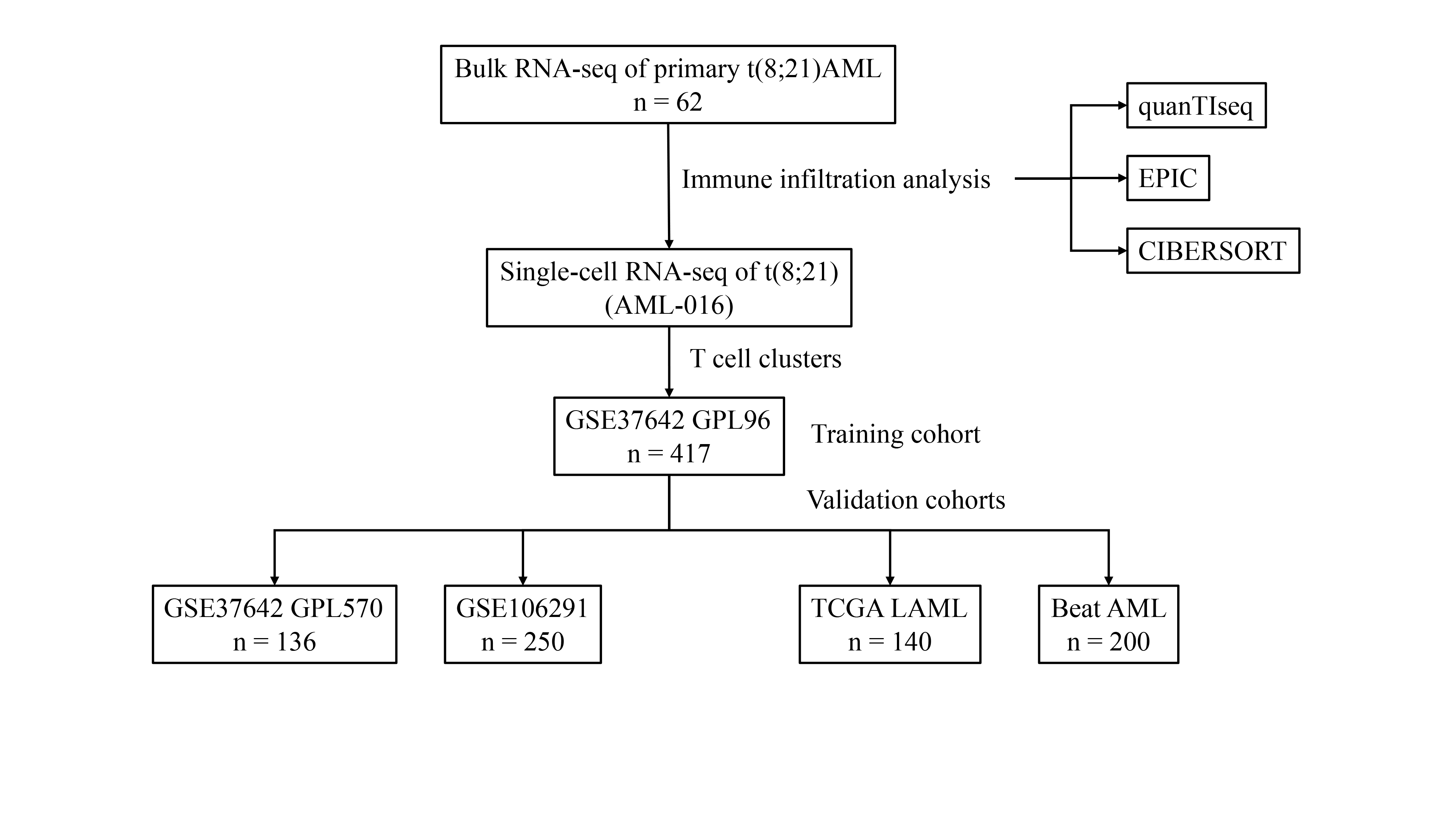

Supplement: Supplementary Figure 1 — Overview of the study design. [file Image_1.tif]

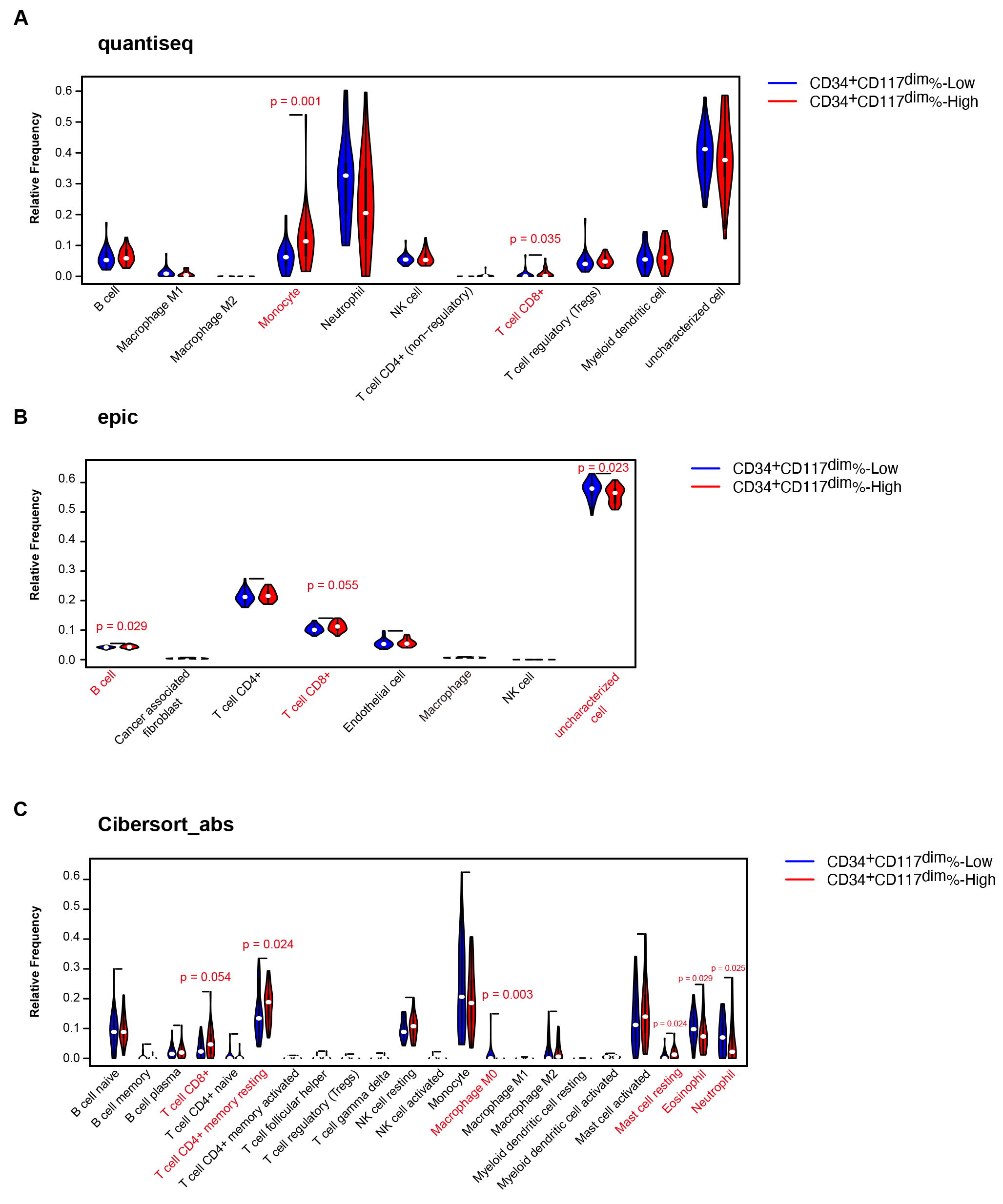

Supplement: Supplementary Figure 2 — Infiltrating immune cells in the BM microenvironment of t(8;21) AML patients. Bar plot showing the comparison of inferred infiltrated immune cells between the two subgroups, namely CD34+CD117dim%-High subgroup and CD34+CD117dim%-Low subgroup, through quanTIseq (A), EPIC (B) and CIBERSORT (C). Statistical analysis was compared with a two-sided Wilcoxon rank-sum test. [file Image_2.tif]

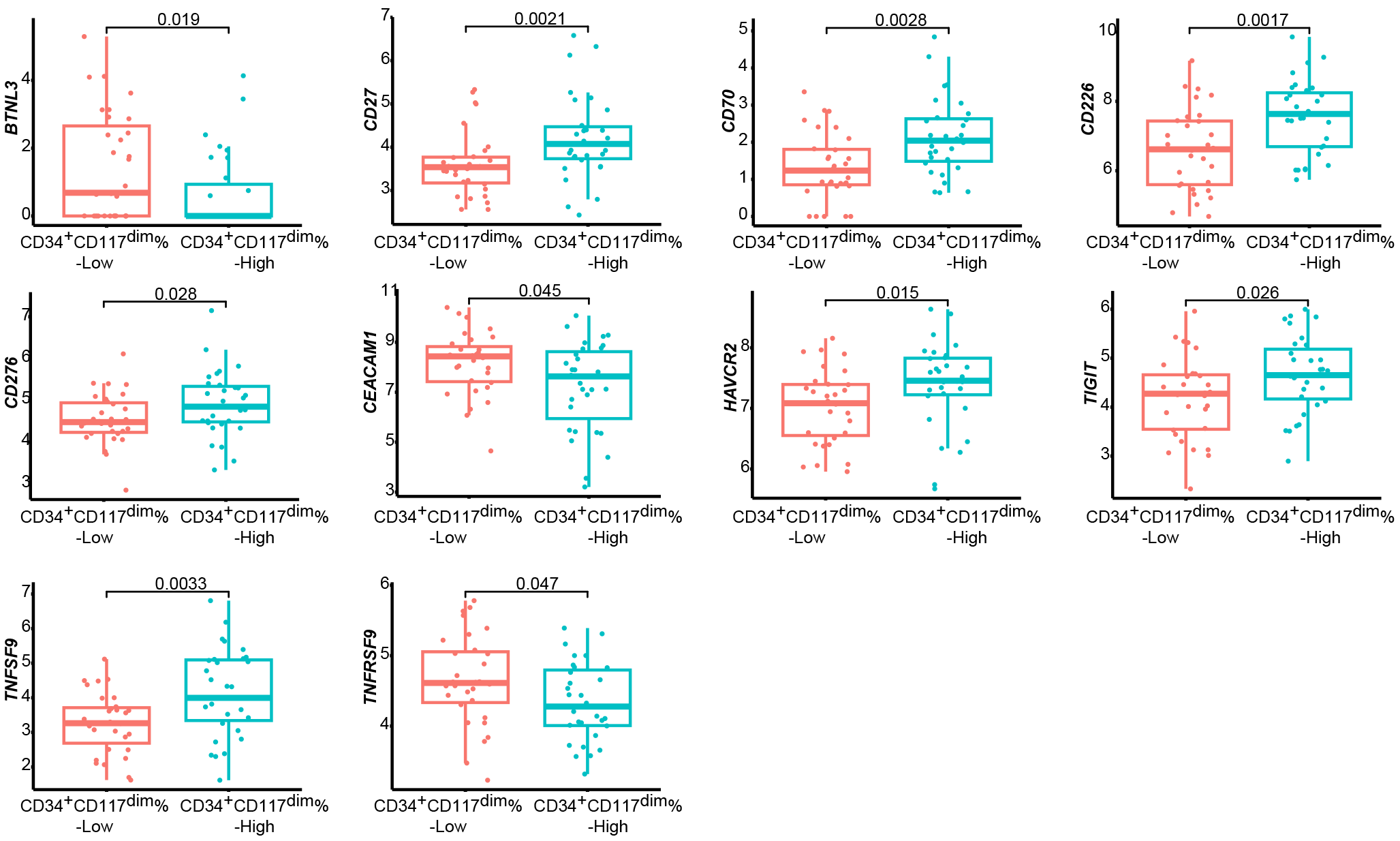

Supplement: Supplementary Figure 3 — Differences in the expression of immune checkpoint genes between the two subgroups, namely CD34+CD117dim%-High subgroup and CD34+CD117dim%-Low subgroup. Statistical analysis was compared with a two-sided Wilcoxon rank-sum test. [file Image_3.tif]

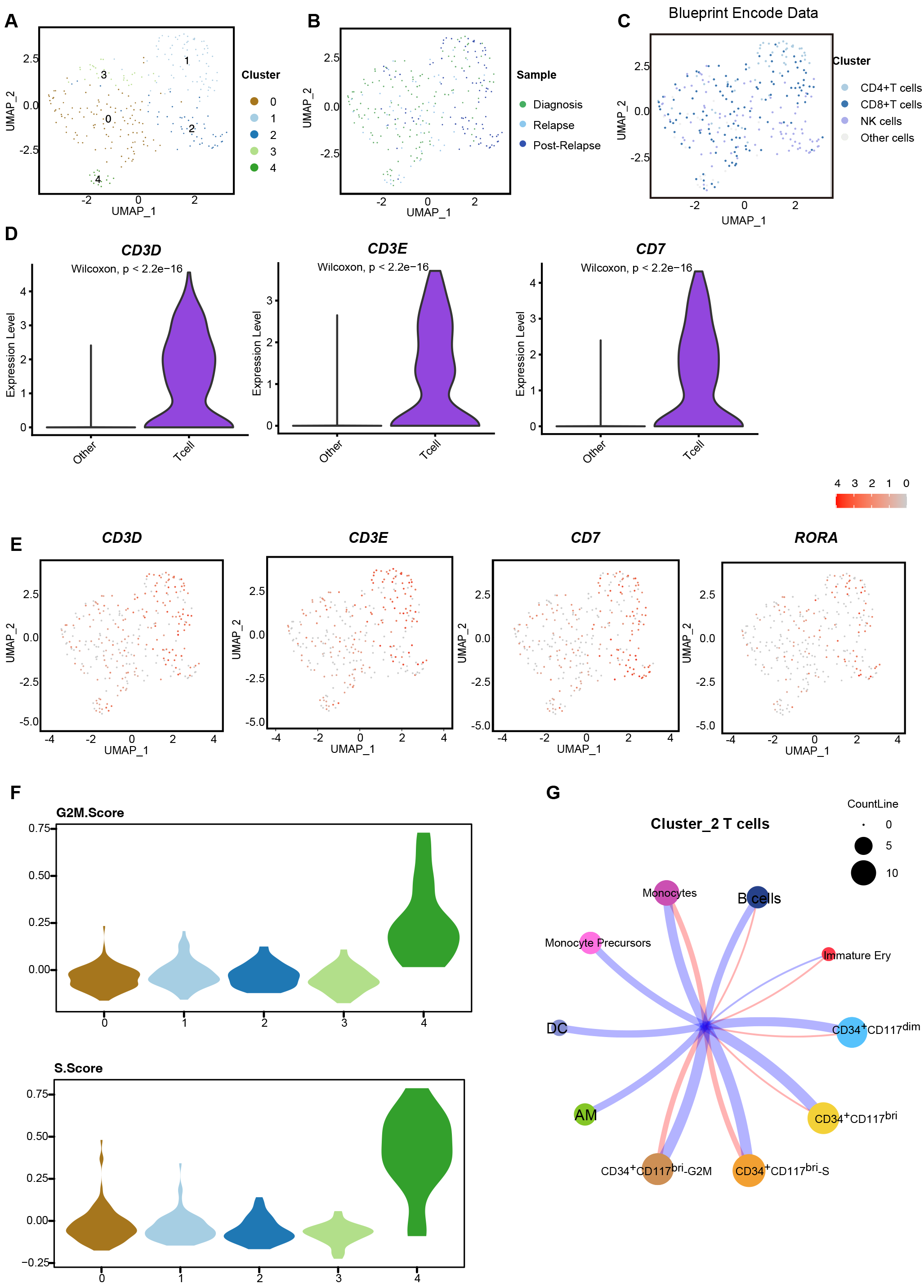

Supplement: Supplementary Figure 4 — Single-cell RNA-seq atlas of T cells across disease states in the AML-016 cohort. (A, B). Uniform manifold approximation and projection (UMAP) plot of T cells analyzed via scRNA-seq and integrated across all samples after removal batch effects. (A) T-cell clusters annotated with different colors. (B) Sample information annotated with different colors. (C) UMAP plot showing annotation results according to the the machine-learning-based software SingleR. (D) Violin plot showing the expression of T-cell markers, including CD3D, CD3E, CD7, between T cells and the rest of BMMCs in AML-016. (E) UMAP plot showing the expression of T-cell markers, including CD3D, CD3E, CD7 and RORA. (F) Violin plot showing the scores of cell cycle-related genes, namely G2M and S scores. (G) CellPhoneDB analysis showed the potential communication of cluster_2 T cells with AML blasts. [file Image_4.tif]

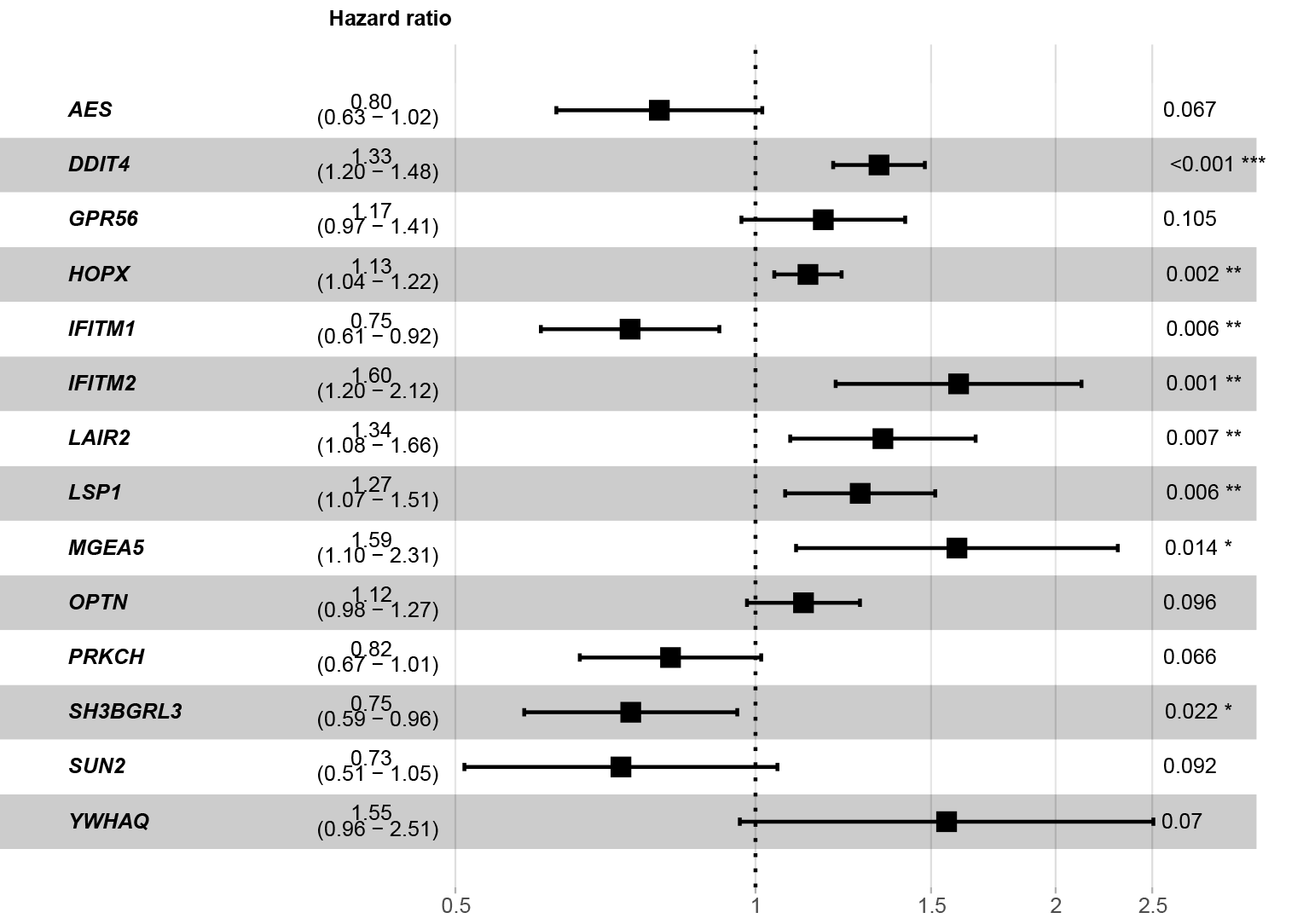

Supplement: Supplementary Figure 5 — Forest plot showing the multivariate analyses in the training cohort (AMLCG 1999, GSE37642_GPL96). HR, hazard ratio. CI, confidence interval. [file Image_5.tif]

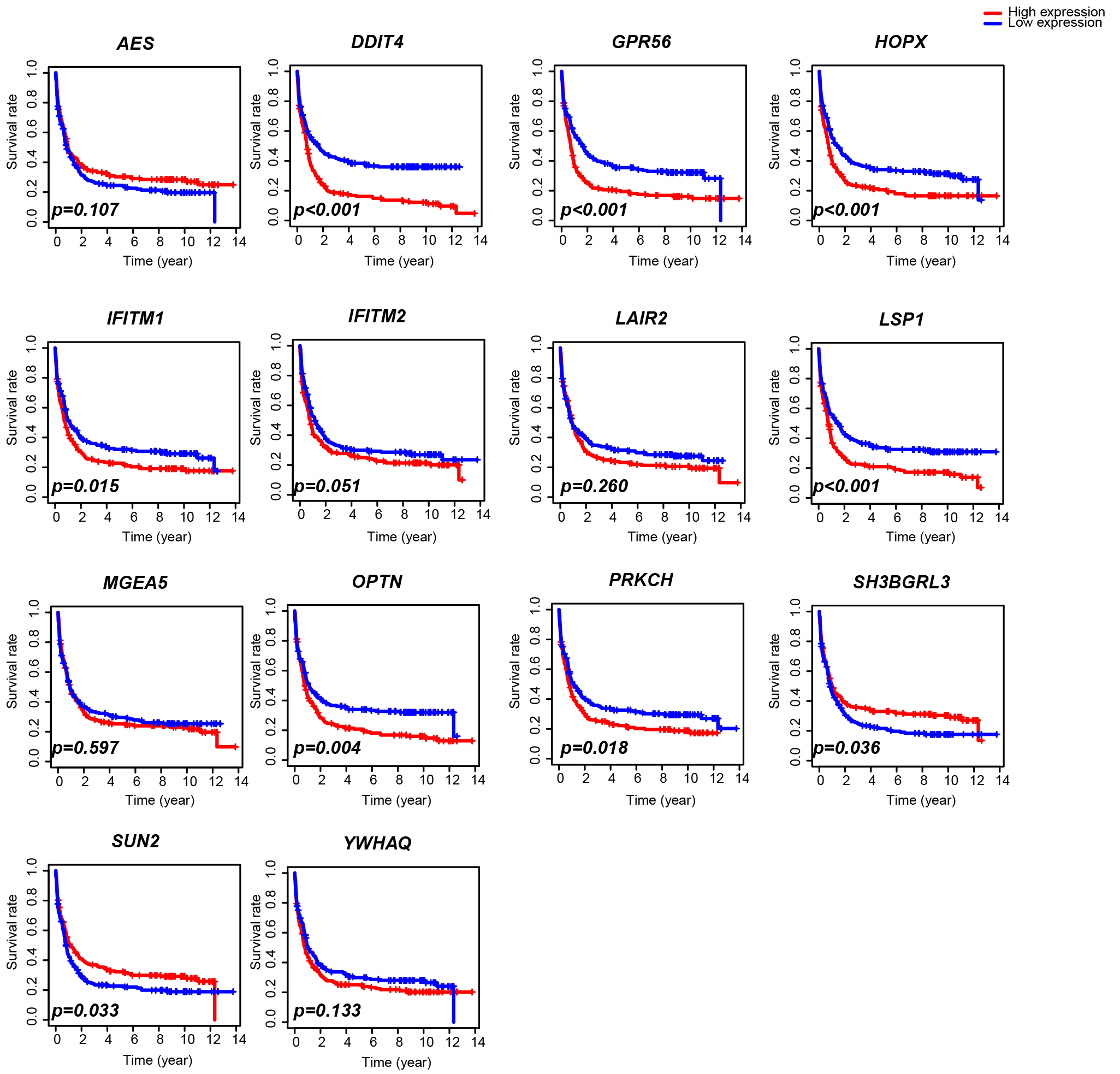

Supplement: Supplementary Figure 6 — Kaplan-Meier curves showed the survival difference stratified by T-cell-related genes in the training cohort (AMLCG 1999, GSE37642_GPL96). Log-rank tests were used to compare the survival differences. [file Image_6.tif]

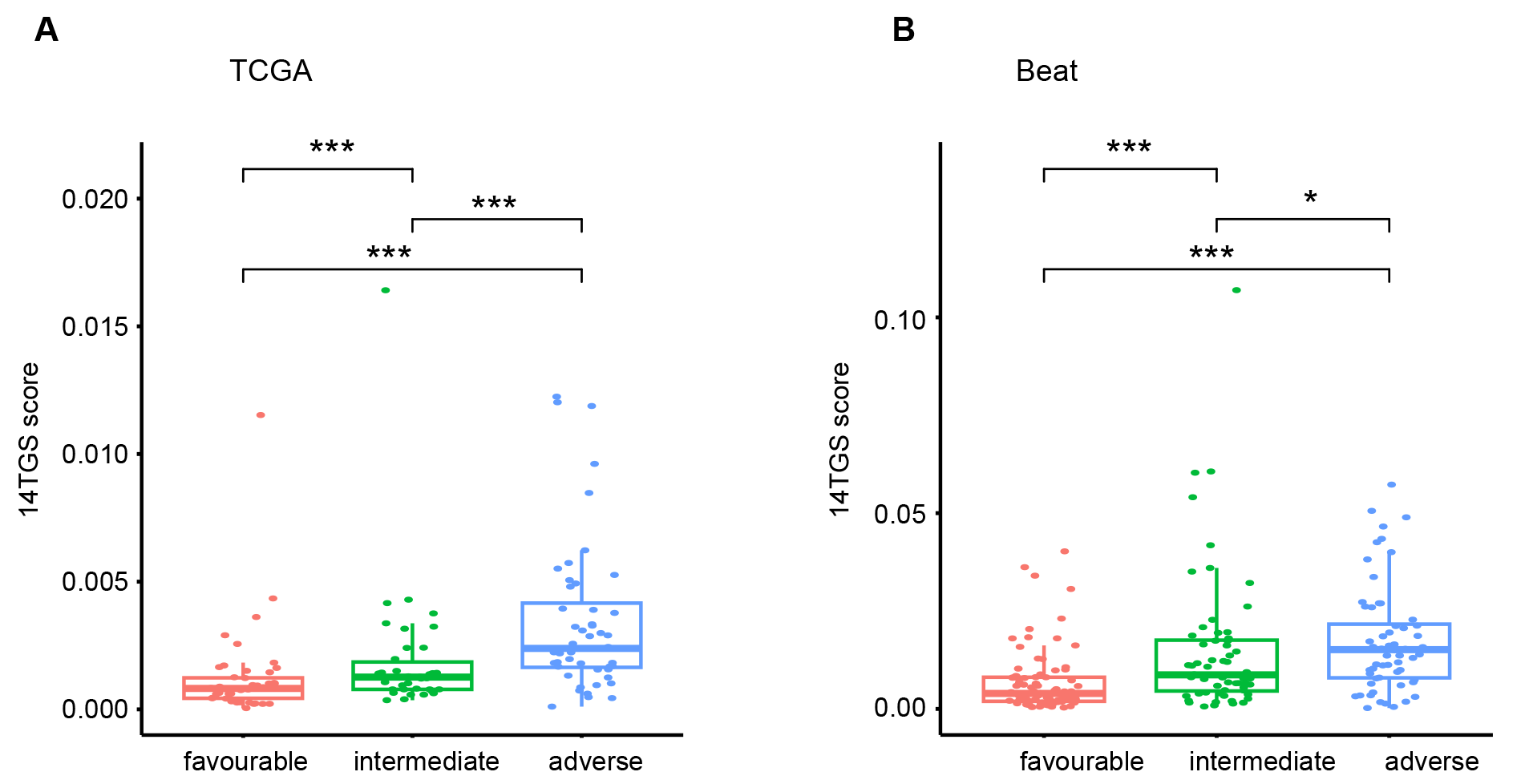

Supplement: Supplementary Figure 7 — Comparsion of 14TGS score among different risk subgroup according to the ELN risk stratification. Wilcoxon test was performed to calculate the statistical differences. *p < 0.05; **p < 0.01; ***p < 0.001. [file Image_7.tif]
